# Adapting, Implementing, and Evaluating Community-Based Dementia Education for Diverse Populations

**DOI:** 10.1093/geroni/igaf122.547

**Published:** 2025-12-31

**Authors:** Jace Flatt, Kristine Talley

## Abstract

In this symposium, we highlight lessons learned in the adaptation, implementation and evaluation of community-based dementia education interventions. First, Dr. Moone highlights lessons learned from the Training to Serve program aimed at improving long-term services and supports (LTSS) providers understanding of LGBTQ+ aging. Using a community-engaged approach, an existing curriculum was adapted for LTSS staff and tailored to LGBTQ+ communities impacted by dementia. The second presentation by Dr. McCarthy highlights the Dementia Friends Information Session—an hour-long session adapted to an online modality during COVID-19 to educate health sciences students on creating dementia friendly communities. This presentation will also include a focus on sustainability of the program. Next, Dr. Nkimbeng shares an educational intervention, African MaDE: Memory and Dementia Education, for African immigrants living with dementia. This presentation describes a community engagement approach used to determine the feasibility and acceptability of African MaDE and efforts to disseminate the program on a larger scale. Finally, Dr. Anderson discusses the adaptation of the Savvy Caregiver® program for LGBTQ+ caregivers of persons with dementia. This will include lessons learned from the use of digital and community-engaged research methods and user-centered design approaches to culturally adapted the program for LGBTQ+ caregivers of people with dementia. Dr. Kris Talley an expert in gerontological nursing education and interventions for managing geriatric syndromes and dementia will summarize the conclusions from each speaker’s presentation and place them in the context of efforts needed to advance the adaptation, implementation, evaluation, and dissemination of community-based educational interventions for dementia.

